# In (visual) search for a new distraction: the efficiency of a novel attentional deployment versus semantic meaning regulation strategies

**DOI:** 10.3389/fpsyg.2014.00346

**Published:** 2014-04-28

**Authors:** Gal Sheppes, William J. Brady, Andrea C. Samson

**Affiliations:** ^1^The School of Psychological Sciences/Child Clinical, Tel Aviv UniversityTel Aviv, Israel; ^2^Department of Psychology, New York UniversityNew York, NY, USA; ^3^Department of Psychology, Stanford UniversityStanford, CA, USA

**Keywords:** attention, distraction, visual search, emotion regulation, reappraisal

## Abstract

Cognitive emotion regulation strategies are considered the king’s highway to control affective reactions. Two broad categories of cognitive regulation are attentional deployment and semantic meaning. The basic distinctive feature between these categories is the *type* of conflict between regulatory and emotional processes for dominance, with an early attentional selection conflict in attentional deployment and a late appraisal selection conflict in semantic meaning. However, prior studies that tested the relative efficacy of these two regulatory categories varied the type and the *degree* of conflict. Our major goal was to test the relative efficacy of a novel attentional deployment strategy (visual search distraction) and a classic semantic meaning strategy (reappraisal) that have a different type of conflict but a matched degree of conflict. Specifically, visual search distraction involves a strong degree of attentional selection conflict manifested in attending subtle non-emotional features that are camouflaged within potent negative emotional stimuli. Reappraisal involves a strong degree of appraisal selection conflict manifested in construing neutral reappraisals that rely on potent negative emotional appraisals. Based on our theoretical model we hypothesized and found that visual search distraction was as effective as cognitive reappraisal in down-regulating the experience of low intensity of negative emotion (Study 1), but more effective, less effortful, and more strongly blocking emotional information processing than cognitive reappraisal when regulating high intensity (Study 2). A final study ruled out a demand characteristics explanation by showing that participants’ expectations about how they should feel diverged from how they actually reported feeling following regulation (Study 3). Our findings suggest that the basic difference in the type rather than degree of conflict between attentional deployment and semantic meaning determines strategies’ outcome.

## INTRODUCTION

One of the fundamental questions that attracted early philosophers as well as modern psychologists is how the mind or reason can control basic drives or emotions. For example, Aristotle’s postulation regarding the powerful connection between one’s cognition and subsequent emotional experience has clearly influenced modern theorem in what has become the field of emotion regulation (Gross, 1998a,b, 2013; Koole, 2009; Webb et al., 2012 for reviews). Although emotions can be regulated in many ways other than via cognition, cognitive regulation strategies are highly common in daily life and have been most extensively studied (Parkinson and Totterdell, 1999; Larsen, 2000; Gross, 2014).

According to several conceptual accounts, cognitive emotion regulation involves recruiting deliberate executive control processes that can change emotions at two major stages of information processing – attentional deployment and semantic meaning (e.g., Ochsner and Gross, 2005; Kalisch et al., 2006; Gross and Thompson, 2007; Sheppes and Gross, 2011 2012; Suri et al., 2013 for reviews). A Central feature in these models is that attentional deployment and semantic meaning can be divided into sub-categories (e.g., Ochsner and Gross, 2005, 2008; McRae et al., 2012), that vary in their level of underlying operation overlap. The existence of overlapping sub-categories, raise important questions about relative efficacy and about the validity of the basic distinction between attentional deployment and semantic meaning.

To give one example, consider a central theoretical account that proposed a hypothetical continuum of the relationship between sub-strategies of attentional deployment and semantic meaning (Ochsner and Gross, 2005). In this continuum the distance between an attentional deployment sub-strategy and a semantic meaning sub-strategy denotes relative reliance on overlap in neural and cognitive processes. In plain words an attentional deployment sub-strategy that is located close on the continuum to a semantic meaning sub-strategy has more process overlap relative to two sub-strategies that are further apart.

While clearly influential, the hypothetical continuum suggested by Ochsner and Gross (2005) does not provide direct predictions or empirical evidence regarding expected efficacy among different sub-categories of attentional deployment and semantic meaning. Furthermore, as will be shown below, prior empirical investigations tested attentional deployment sub-strategies that have minimal process overlap with semantic meaning strategies. Accordingly, the major goal of the present study was to extend our conceptual framework (Sheppes and Gross, 2011, 2012) to predict and test the relative efficacy of a novel attentional deployment sub-strategy that has considerable underlying process overlap with a classic semantic meaning sub-strategy.

The starting point of our conceptual framework is that limited cognitive capacity poses processing constraints that result in a competition or conflict between emotional and regulatory processes for dominance over behavior. Drawing upon major information processing theories (e.g., Pashler, 1998; Hübner et al., 2010) and the process model of emotion regulation (Gross and Thompson, 2007) we argue that deliberate cognitive regulation starts with a top-down generated goal to influence an emotion (Gross et al., 2011a,b), that can be achieved via early disengagement from emotional processing at an attention deployment stage, or via an engagement with emotional processing that is modulated at a late semantic meaning stage.

The representative attentional deployment sub-strategy we investigated in prior studies is distraction (e.g., Van Dillen and Koole, 2007), which involves an early attentional selection *type* of conflict between regulatory and emotional processes. Specifically, distraction involves producing neutral thoughts that compete with emotional information at an early selective attention stage. In distraction there is also a minimal *degree* of conflict between regulatory and emotional processes because the neutral thoughts that are formed are independent from emotional information (see similar arguments regarding a “production function” for distraction in Kalisch et al., 2005). The representative semantic meaning sub-strategy we studied is reappraisal (Gross, 2002), which involves a late appraisal selection type of conflict. Specifically, reappraisal involves forming neutral reinterpretations that compete with emotional appraisals at a late semantic meaning stage. In reappraisal there is also a strong degree of conflict between regulatory and emotional processes because the neutral reinterpretation semantically depends on the original emotional appraisal (see similar arguments regarding a “monitoring function” for reappraisal in Kalisch et al., 2006).

According to our conceptual framework, these underlying characteristics of distraction and reappraisal result in a differential cost-benefit efficacy tradeoff (Sheppes and Gross, 2011, 2012). Specifically, in distraction the benefit of disengaging from emotional information early, is manifested in successful modulation of high emotional intensity information (e.g., Sheppes and Meiran, 2007, 2008; Sheppes et al., 2009; Thiruchselvam et al., 2011). However, distraction’s major costs involve memory impairments of emotional information, and impaired long term adaptation that in many cases requires attending and making sense of emotional events (Wilson and Gilbert, 2008 for a review). By contrast, in reappraisal the engagement with elaborated semantic processing prior to late modulation, is manifested in less successful modulation of high intensity emotional information (e.g., Kanske et al., 2011; Paul et al., 2013; Schönfelder et al., 2013). Nevertheless, the major benefit of engaging with emotional information via reappraisal involves intact memory of emotional information and long-term adaptation (e.g., MacNamara et al., 2011; Blechert et al., 2012).

Although our framework clearly describes the cost-benefit profile of distraction and reappraisal, these two sub-categories of attentional deployment and semantic meaning have minimal process overlap, because they vary on the type and degree of conflict between regulatory and emotional processes. In the present study, we wanted to provide a more stringent test of the differential efficacy of the two major attentional deployment and semantic meaning regulation categories. We wished to maintain the differential type of conflict in attentional deployment and semantic meaning, but to increase the degree of conflict in attention deployment so that it would resemble the high degree of conflict in semantic meaning. Specifically, we wished to create a regulatory attentional selection option that involves a strong *degree* of attentional selection conflict that resembles the strong degree of semantic meaning conflict in reappraisal (and which is higher than the weak degree of attentional selection conflict in the classic attentional distraction).

Augmenting the degree of conflict in attentional deployment requires increasing the attentional selection competition between the emotional and regulatory process. Regulatory attentional deployment involves selecting a neutral aspect of an emotional situation to focus on. While emotional situations can be internal or external, the most controlled way to induce emotions involves using external emotional situations or stimuli (e.g., using emotional pictures). For this reason, we decided to increase the degree of external attentional selection competition, via stimulus based competition. Specifically, we developed a task that involves performing continuous visual search for subtle non-emotional features (i.e., small letters that were camouflaged) in emotional pictures. The strong degree of conflict is between a top down regulatory goal to direct attention to a visual search for subtle letter stimuli and between a salient attention grabbing emotional process. Conceptually our visual search distraction is located at the attention deployment end (i.e., “attention to non-emotional stimulus attributes”) that is closest to semantic meaning in Ochsner and Gross’s (2005) model, thus reflecting considerable process overlap.

The degree of stimulus based competition in our visual search distraction can be differentiated from prior studies that used other forms of distraction. Prior to selectively reviewing these prior studies it is important to note that there are numerous conceptual accounts and supporting studies on the interactions between cognition and emotion (see Robinson et al., 2013 for a systematic coverage). These can be divided into two broad categories that differ in the examined directionality of the relationship between emotion and cognition. One category involves studying how emotions affect cognition (e.g., Pessoa, 2009; Iordan et al., 2013; Okon-Singer et al., 2013 for reviews). While clearly influential and important, a second category that involves studying how cognition affects emotion is more directly related to emotion regulation and accordingly to the present study. Specifically, emotion regulation is most clearly defined when there is a goal to utilize cognition in order to modulate emotion (see Ochsner and Gross, 2005; Gross et al., 2011a,b), and supporting studies examine how cognitive regulatory strategies influence emotional responding. Accordingly, we limit our review to studies that can be positioned under an emotion regulation umbrella. Specifically, in studies that presented letters or numbers as regulatory distractions prior to emotional picture presentation (e.g., McRae et al., 2010; Van Dillen et al., 2013), there is no stimulus based competition because symbols are represented and maintained in working memory before the emotional stimulus is introduced. In studies that ask participants to regulate by fixing their gaze at a non-emotional part of an emotional stimulus (e.g., Dunning and Hajcak, 2009; Urry, 2010) there is a minor stimulus based competition, because the attended percept is non-emotional. Last, studies that induce regulation by presenting a mathematical equation during emotional picture presentation (e.g., Kanske et al., 2011; Schönfelder et al., 2013) involve initial stimulus based competition during encoding but no stimulus based competition during actual performance. It is important to note that in all of these studies as well as in our visual search distraction successful disengagement is expected, because regulatory and emotional tasks do not share task relevance (see Lichtenstein-Vidne et al., 2012 for a relevant discussion).

The major goal of the present study was to compare the relative efficacy of a novel attentional deployment strategy that has considerable underlying process overlap with a classic semantic meaning sub-strategy. According to our conceptual framework, the type as well as degree of conflict between regulatory and emotional processes can determine regulatory efficacy. In the present study, although the degree of conflict in visual search distraction was set to resemble the degree of conflict in reappraisal, visual search distraction still blocks emotional information at an earlier processing stage relative to reappraisal. We therefore expected that visual search distraction would be more effective than reappraisal under increased emotional challenge – high emotional intensity. Converging support for the expected effectiveness of attentional deployment despite enhanced degree of cognitive conflict, comes from studies showing that attentional selection strategies that involve high cognitive load result in successful modulation of high intensity information (e.g., Pessoa et al., 2002; Erk et al., 2007; Van Dillen and Koole, 2007; Van Dillen et al., 2009).

In the present paper we report three studies that tested the relative efficacy of visual search distraction to reappraisal. In Study 1 we tested for the first time visual search distraction and compared its efficacy to reappraisal when regulating low negative emotional intensity. Based on our conceptual framework (Sheppes and Gross, 2011, 2012) we predicted both strategies to be equally efficacious in modulating subjective negative ratings and in perceived effort. In Study 2 we examined the relative efficacy and underlying operation of both regulatory conditions under low and high intensity situations. Extending our conceptual framework we expected that under high (but not low) emotional intensity visual search distraction would be more efficacious than reappraisal as manifested in negativity and difficulty ratings. We also employed two performance-based measures to evaluate underlying operation of both strategies. Specifically, we examined the online stimulus based competition in visual search distraction, and basic differential processing in both strategies. We expected that online stimulus based competition in visual search distraction would be evident in showing that letter count performance is influenced/reduced by high relative to low emotional intensity (see Sheppes et al., 2014, Study 5 for related findings). We also expected that the disengagement nature of visual search distraction and the engagement nature of reappraisal would be evident in impaired memory of emotional contents for visual search distraction relative to reappraisal (Sheppes and Meiran, 2007, 2008; Sheppes et al., 2011). In Study 3 we wished to show that the relative efficacy results obtained with self reports cannot be fully explained by demand characteristics. To that end we predicted that participants’ *expectations* about how they should feel would diverge from how they *actually* feel while implementing visual search distraction.

## STUDY 1: TESTING VISUAL SEARCH DISTRACTION AND REAPPRAISAL UNDER LOW EMOTIONAL INTENSITY

Study 1 aimed to test the efficacy of visual search distraction for the first time and to compare it to reappraisal in the context of low emotional intensity. Based on our framework showing similar efficacy between attentional deployment and semantic meaning for low emotional intensity stimuli, visual search distraction was expected to be as effective and difficult to implement as reappraisal (Sheppes and Gross, 2011, 2012).

The experiment used a within-subjects design with five different conditions that varied trial by trial: “look” and “search” for both, negative and neutral pictures, and “reappraise” for negative pictures only. In the visual search distraction condition, participants were instructed to focus on letters embedded within negative or neutral picture and to search for as many letters as they could find. In the “look” conditions participants were instructed to attend to the negative or neutral stimuli and to freely allow themselves to experience feelings. This commonly used instruction (c.f. Coan and Allen, 2007 for a review) allowed obtaining a control condition that isolates emotion generation from spontaneous regulation efforts. In the “reappraise” condition participants were instructed to attend to emotional stimuli but to focus on a specific aspect of the image they could think differently about in order to change the meaning in a way that would make them feel less negative. For example, participants were told that they could think about how the problem or outcome depicted in the image might soon be resolved.

### METHOD

#### Participants

Twenty-two participants (19 female, 3 male) with an age range of 18 to 29 (*M* = 22.20, SD = 2.91) identified themselves as Caucasian (27.3%), Asian (18.2%), Latino (18.2%), African-American (9.1%), Native American (9.1%), Middle Eastern (4.5%), Mixed (4.5%) and 9.1% declined to provide the information. All participants were recruited at a West coast University: three of the participants received course credit and 19 got compensation ($10 awarded).

#### Stimuli

There were 25 images for each of the five conditions. Seventy-five low negative pictures (valence: *M* = 3.03, SD = 0.78; arousal: *M* = 5.60, SD = 0.79) for the conditions “look negative,” “reappraise negative,” and “search negative” and 50 neutral pictures (valence: *M* = 5.14, SD = 0.73; arousal: *M* = 3.14, SD = 0.82) were used for the conditions “look neutral” and “search neutral” from the International Affective Pictures System (IAPS; Lang et al., 2008).

**FIGURE 1:**
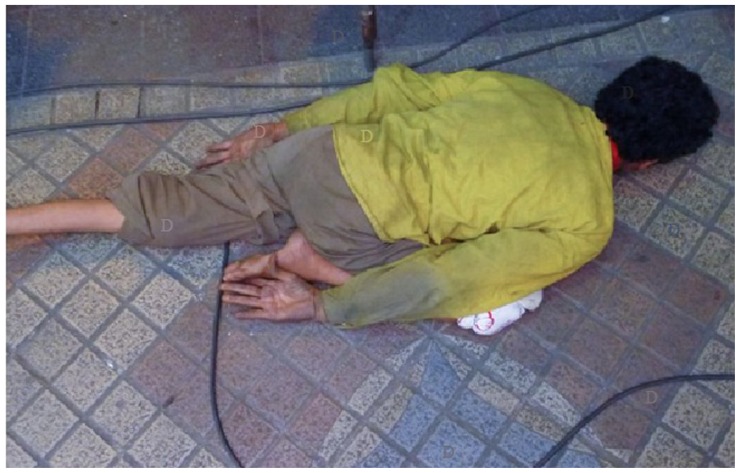
**An example of picture used for the “search” negative condition.** In this trial participants were asked to count as many “D”s as they can. Letters were superimposed on the original picture.

To allow for random assignment of pictures to conditions, each picture existed in an unmodified version for the “look” and “reappraise” conditions and in a modified version with letters superimposed on the picture for the “search” conditions. The images were edited in order to embed 10 to 14 letters (24pt sized) at multiple locations using Adobe Photoshop (see **Figure 1** for an example). The color of each letter varied based on the color of the placement area in the picture in order to slightly camouflage the letters within the image in an effort to increase the stimulus-based competition. This way, the letters slightly blended in with their placement spot on the picture yet were still visually detectable. An initial pilot study was conducted to ensure that the detection of the letters in the pictures were of approximate equal difficulty. With *N* = 19 it was measured whether it was possible to count the majority of letters within a 8-s time window which was used for the actual experiment. Depending on performance, maximum ±2 letters were deleted or added, resulting in 10.44 letters per picture on average (SD = 1.05).

#### Procedure

After receiving participant’s consent, participants were first taught what each strategy means. Participants were instructed to attend naturally to the picture and let their feelings and thoughts occur naturally (“look” condition), to reinterpret the meaning of the stimulus such that they might feel less negative about it (“reappraisal” condition; see Sheppes et al., 2014 for exact wording), or to search for as many letters they can find embedded in the picture (“search” condition). The rating scales were explained and how to report the number of letters they counted. Following the teaching part participants completed five practice trials that were identical to the actual experiment. In the actual experiment 25 pictures were randomly assigned to each of the five conditions. The presentation of the trials was randomized with maximally two of the same instructions in a row. Each trial started with a 3 s long fixation cross, followed by a slide showing the instruction [“look,” “reappraise,” or “search for [letter],” with an indication of the letter to be searched for in the specific trial]. Each picture was presented for 8 s, followed by rating scale for negativity ranging from 1 (“not negative at all”) to 5 (“very negative”) and difficulty ranging from 1 (“not difficulty at all”) to 5 (“very difficult”) that were each shown until the participant responded. In the “search” condition, participants were also asked to type the number of letters they counted. The whole experiment lasted approximately 45 min.

### RESULTS AND DISCUSSION

#### Negativity

**FIGURE 2:**
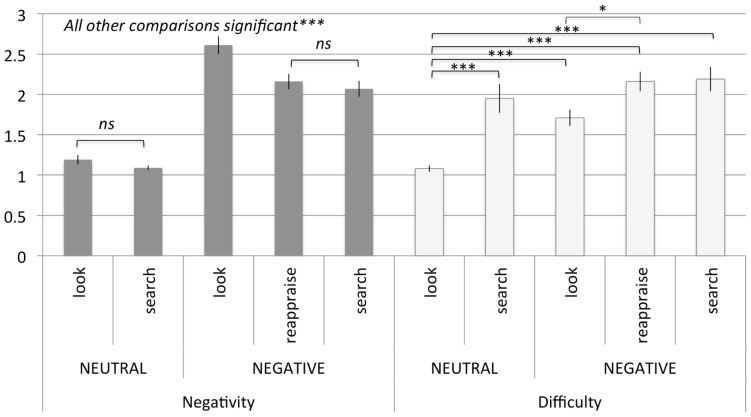
Study1: averages and standard errors of negativity (gray bars) and difficulty ratings (white bars) for the different instruction conditions for neutral and negative pictures (*N* = 22). *p <0.05, **p < 0.01, ***p < 0.001.

A within subject one-way ANOVA was conducted to determine whether the conditions differed in negativity. Mauchly’s test indicated that the assumption of sphericity had been violated [χ^2^(9) = 20.86, *p* = 0.014]; therefore degrees of freedom were corrected using Greenhouse-Geisser estimates of sphericity (ε = 0.68). The main effect was significant, indicating that the conditions differed from each other [*F*(2.72,57.09) = 113.39, *p* <0.001, ω^2^ = 0.34]. Central to our predictions, post hoc pairwise comparisons with Bonferroni adjustment for multiple comparisons revealed that while the two regulatory forms of “search negative” (*M* = 2.07, SD = 0.47) and “reappraise negative” (*M* = 2.16, SD = 0.46) were not significantly different from each other [*F*(1,21) = 2.12, *ns*], both regulatory forms were associated with reduced negativity relative to the control “look negative” condition [*M* = 2.61, SD = 0.53; “search negative” vs. “look negative”: *F*(1,21) = 34.23, *p* <0.001, “reappraise negative” vs. “look negative”: *F*(1,21) = 33.26, *p* <0.001]. Complete details of all conditions are presented in **Figure 2**. This pattern of results indicates that the two emotion regulation conditions were equally effective in reducing negative emotions, and confirms our hypothesis that visual search distraction can down-regulate negative emotions with as much efficacy as reappraisal for low intensity negative images. 

#### Difficulty

A within subject one-way ANOVA was conducted to determine whether the conditions differed in difficulty. Mauchly’s test indicated that the assumption of sphericity had been violated [χ^2^(9) = 34.44, *p* <0.001]; therefore degrees of freedom were corrected using Greenhouse-Geisser estimates of sphericity (ε = 0.55). There was a significant effect of difficulty [*F*(2.18,45.78) = 19.73, *p* <0.001, ω^2^ = 0.17]. Follow up analyses with Bonferroni adjusted single comparisons revealed that the two regulatory forms “search negative” (*M* = 2.19, SD = 0.72) and “reappraise negative” (*M* = 2.17, SD = 0.59) were equally difficult to employ [*F*(1,21) <1]. In addition, regulatory “Reappraise negative” resulted in more perceived difficulty relative to the control “look negative” condition [*M* = 1.71, SD = 0.50; *F*(1,21) = 12.76, *p* = 0.018]. Unexpectedly, regulatory “search negative” was not significantly different from the control “look negative” condition [*F*(1,21) = 9.21, ns]. Results of all conditions are presented in **Figure 2**. These results confirmed our expectation that visual search distraction and reappraisal were not significantly different in difficulty in low negative images. Somewhat unexpectedly, we found that applying reappraisal, but not visual search distraction, was perceived as more difficult than naturally attending to pictures. However, it is important to mention that both visual search distraction and reappraisal averaged toward the “not difficult at all” side of the scale.

#### Search accuracy

Given that we developed a novel visual search distraction task, it was important to show that participants perform it adequately. Three lines of evidence suggest that this was indeed the case. First, on average, participants showed moderately high performance rates with 66.95% accuracy [on average participants counted 6.99 (SD = 2.27) out of 10.44 actual letters]. Second, across participants there was a significant positive correlation between the number of counted letters and the actual number of letters *r*(1100) = 0.37, *p* <0.001. Third, counting accuracy correlated negatively with difficulty ratings in both conditions [negative pictures: *r*(550) = -0.12, *p* = 0.004, neutral pictures: *r*(550) = -0.16, *p* <0.001].

## STUDY 2: TESTING VISUAL SEARCH DISTRACTION AND REAPPRAISAL UNDER LOW AND HIGH EMOTIONAL INTENSITY

Findings from Study 1 indicated that visual search distraction is equally efficacious emotion regulation strategy as reappraisal when modulating low negative intensity information. Study 2 was conducted in order to extend our prior finding in two important ways. The first goal was to provide initial evidence for differential efficacy under increased emotional challenge. Specifically, according to our conceptual framework (Sheppes and Gross, 2011, 2012) early disengagement from emotional processing via attentional deployment should more successfully modulate high intensity emotional responses relative to late modulation via semantic meaning. We therefore expected that under high intensity visual search distraction would result in reduced negativity and difficulty relative to reappraisal. In addition, we also expected to replicate findings from Study 1 by showing that under low intensity reappraisal and visual search distraction are equally effective. Our second goal was to test the underlying operation of the two regulatory conditions using two performance-based measures. Specifically, in order to demonstrate that visual search distraction involves a stimulus based competition between the regulatory and emotional processes, we measured letter search performance for high relative to low intensity emotional information. Demonstrating that performance is reduced in high relative to low intensity would show that emotional information processing conflicts with the top-down regulatory process. Furthermore, to demonstrate the basic disengagement nature of distraction and basic engagement nature of reappraisal we conducted a surprise memory test of emotional contents and predicted that memory will be impaired for visual search distraction relative to reappraisal (Sheppes and Meiran, 2007, 2008; Sheppes et al., 2011). To that end, the present study maintained the use of the three instructions (“look,” “reappraise,” and “search”) that were tested in two negative emotional intensities (low and high).

### METHOD

#### Participants

Forty-four participants between the ages of 18 and 49 (*M* = 23.50, SD = 7.45, 22 females) identified themselves as Caucasian (43.2%), Latino (18.2%) Asian or Pacific Islander (15.9%), mixed (9.1%), African-American (6.8%), and 6.8% as other. All participants were recruited from West Coast Universities and got compensation with $10 (*N* = 20) or received course credit (*N* = 24) for the participation in the experiment, which lasted approximately 70 min.

#### Stimuli

Two new sets of pictures were selected from the IAPS (Lang et al., 2008), both including 45 negative pictures of two intensities (Sheppes et al., 2011, 2014). One set consisted of low negativity pictures (valence: *M* = 3.53, SD = 0.42; arousal: *M* = 5.22, SD = 0.78), the other were highly negative (valence: *M* = 1.95, SD = 0.31; arousal: *M* = 6.08, SD = 0.74). To ensure random assignment of pictures to conditions each picture had an edited version for the “search” condition to have 10 to 14 (*M* = 10.81, SD = 1.27) 24pt letters embedded using Adobe Photoshop.

#### Procedure

***Emotion regulation task.*** Study 2 maintained the same event-related design, randomization rules, sequencing of consenting, instructions, picture presentation, and ratings, as in Study 1. There were six conditions (three instructions by two intensity levels) with 15 stimuli per condition, resulting in a total of 90 trials. After the main experiment, a surprise memory test was introduced.

***Visual search performance.*** Following each trial of the Search condition participants indicated how many letters they counted.

***Surprise memory task.*** On each of the 90 trials of the memory test, participants were presented with two pictures (see also Sheppes et al., 2011 for a similar test). Participants were told that the two pictures differed only marginally and they should decide which of the pictures they had seen in the previous portion of the experiment (Instructions: “Throughout the first part of our study, you saw a series of pictures. Next, you will be presented with a set of pairs of pictures. For each pair of pictures, there is a key difference between the pictures. You will always be given a hint, telling you what the difference is between the pictures. Please select which one of the pictures you saw during the previous experiment”). One picture had been presented during the actual experiment (e.g., a picture of a crying baby); the other picture was a Photoshop-modified version of the same picture. In half of these modified pictures, a central emotional feature had been added (e.g., the baby had extra tears); in the other half, a central emotional feature had been excluded (e.g., the baby was missing a few tears). Above the two pictures, a keyword pertaining to the difference between the pictures was presented (e.g., “tears”).

### RESULTS AND DISCUSSION

#### Negativity

**FIGURE 3:**
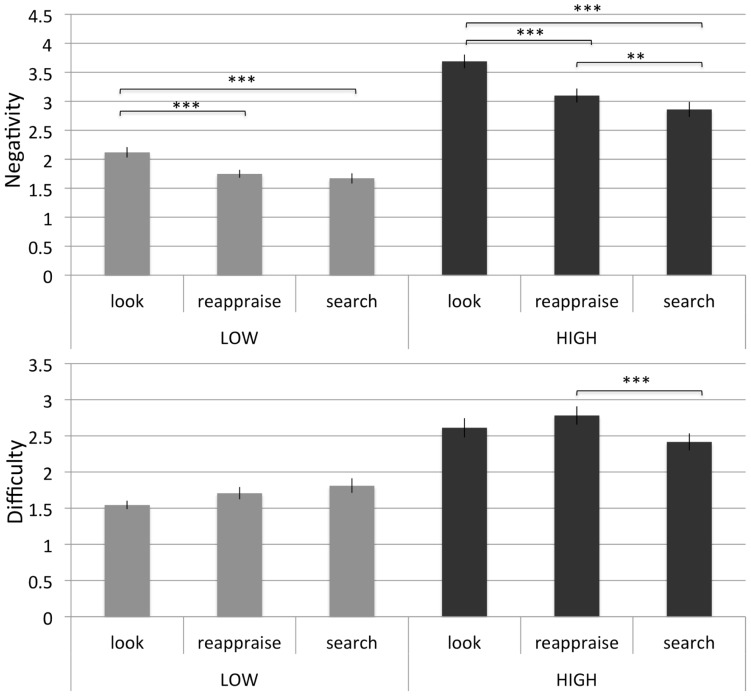
Study 2: mean of negativity and difficulty ratings in low and high negative pictures in the three conditions (“look,” “reappraise,” and “search”), *N* = 44. *p <0.05, **p <0.01, ***p <0.001.

A 2 × 3 repeated measures ANOVA was carried out to examine the interaction between intensity (low vs. high negativity images) and instruction type (“look,” “search,” and “reappraise”). The main effects of intensity [*F*(1,43) = 316.93, *p* <0.001] and of instruction type were significant [*F*(2,86) = 43.60, *p* <0.001] indicating that the negativity of the pictures, as well as the three instruction conditions had an effect on negativity ratings. These main effects were qualified by the expected interaction [*F*(2,86) = 11.00, *p* <0.001]. Replicating findings from Study 1, under low intensity, the two regulatory forms “search” (*M* = 1.67, SD = 0.62) and “reappraise” (*M* = 1.75, SD = 0.46) did not differ from each other [*F*(1,43) = 2.02, *ns*] but were both associated with lower negativity than the control “look” condition [*M* = 2.12, SD = 0.62; “search” vs. “look”: *F*(1,43) = 27.07, *p* <0.001, “reappraise” vs. “look”: *F*(1,43) = 27.77, *p* <0.001]. Importantly, confirming our predictions for high intensity, while both regulatory forms “reappraise” (*M* = 3.10, SD = 0.83) and “search” (*M* = 2.86, SD = 0.85) were associated with lower negativity than the control “look” condition [*M* = 3.70, SD = 0.80; “reappraise” vs. “look” *F*(1,43) = 39.29, *p* <0.001; “search” vs. “look” *F*(1,43) = 57.08, *p* <0.001], regulatory “search” was also associated with lower negativity relative to regulatory “reappraisal” [*F*(1,43) = 13.22, *p* = 0.002, see **Figure 3**].

#### Difficulty

Difficulty scores were subjected to a 2 × 3 repeated measurement ANOVA to analyze the interaction between intensity and instruction type. While the intensity revealed a significant main effect [*F*(1,43) = 129.89, *p* <0.001], instruction type did not [*F*(2,86) = 1.99, *ns*]. However, the expected interaction was significant [*F*(2,86) = 18.18, *p* <0.001] in that the instruction conditions affected difficulty ratings dependent on the intensity of the pictures. Follow up analyses with Bonferroni adjusted single comparisons revealed that the only reliable difference indicated increased difficulty of regulatory “reappraise” [*M* = 2.78, SD = 0.85] relative to the control “search” condition [*M* = 2.42, SD = 0.80] in the high negativity condition [*F*(1,43) = 23.33, *p* <0.001]. Information regarding all conditions appears in **Figure 3**. These results provide complementary evidence to those obtained with negativity ratings in showing that visual search distraction required less cognitive effort (and was thus more efficacious) than reappraisal for high intensity stimuli.

#### Search accuracy

Percent correct of the number of counted letters per condition was computed. Confirming our predictions regarding a stimulus based competition between regulation and emotional processes in visual search distraction, a paired *t*-test revealed that accuracy in the “search high” condition (*M* = 72.39%, SD = 10.34) was lower than in the “search low” condition [*M* = 74.47%, SD = 9.61; *F*(1,43) = 5.09, *p* = 0.029]. In addition, finding that emotional intensity affected visual search performance is congruent with a top-down generated regulatory goal that cannot completely block emotional processing.

#### Memory accuracy

Percent correct of memory performance was computed. The 2 × 3 repeated measurement ANOVA showed that there was only a main effect for instruction type [*F*(2,84) = 16.79, *p* <0.001]. As expected, regulatory “reappraise,” which involves engagement with emotional information processing, resulted in the highest memory scores (*M* = 65%, SD = 7) which was better than the control “look” condition [*M* = 61%, SD = 11; *F*(1,42) = 4.06, *p* = 0.05] and regulatory “search” (*M* = 54%, SD = 10) that involves disengagement from emotional processing [*F*(1,42) = 43.60, *p* <0.001]. Regulatory “Search” also resulted in worse memory relative to the control “look” condition [*F*(1,42) = 11.29, *p* = 0.002].

## STUDY 3: RULING OUT DEMAND CHARACTERISTICS EXPLANATION FOR DIFFERENTIAL EFFICACY

Findings from Study 2 supported our conceptual account by showing that participants reported experiencing less negative and lower perceived difficulty when performing visual search distraction relative to reappraisal under high emotional intensity. While encouraging, these results were obtained using subjective self report measures that are susceptible to demand characteristics. Specifically, participants may not *actually* feel more negative following reappraisal relative to visual search distraction, rather they think that they are *expected* to feel more negative following reappraisal and this knowledge governs their self reports.

One powerful method to rule out this alternative explanation is to measure how participants think they are expected to feel toward different strategies, independent from their actual emotional experience while employing these strategies. Demand characteristics concerns can be minimized if participants’ expectations about how they should feel diverge from how they actually feel.

To achieve this goal we ran a final study where we asked participants to imagine how they think they would feel while employing different regulation strategies under different intensities, without having them actually employ these strategies while being exposed to different emotional intensity stimuli (see Lieberman et al., 2011 for a similar approach). We predicted that self-reports of how participants expect to feel would diverge from those obtained in Study 2 that involved actually employing regulatory strategies.

### METHOD

#### Participants

Forty-one participants with similar characteristics to those in Study 2 were recruited. Specifically, participants were between the ages of 18–49 (24 females) identified themselves as Caucasian (70.7%), Latino (12.2%), Asian or Pacific Islander (4.9%), African American (9.8%), and 2.4% as other. All participants were recruited from Amazon Mechanical Turk and received compensation of $0.50.

#### Procedure

We used a within-subjects 2 × 3 design (Intensity by Instruction Type) as in Study 2 with similar procedure, except that participants in this study were asked to *imagine* employing different conditions (“look,” “reappraise,” “search”) while viewing emotional pictures without actually employing these conditions or actually viewing emotional pictures (Lieberman et al., 2011). Participants first completed practice trials in which they received explanations for what each instruction entailed that matched exactly the instructions given to participants in Study 2. To ensure that participants read and understood how to imagine pictures and to implement each of the conditions, we asked them to type what they imagined for the regulatory conditions during the practice trials.

During the actual experiment, participants *imagined* performing the experimental conditions while viewing different pictures. Instead of actually presenting emotional pictures participants saw verbal descriptions of pictures similar to those presented in Study 2 and got instructions as presented in study 2 (e.g., “Adopt the LOOK instruction while imagining a picture of a MUTILATED HUMAN FACE”). Each participant viewed descriptions of two types of pictures (low and high intensity pictures) for each of the three instructions. After every picture, participants indicated how negative they imagined they would feel, on a scale from 1 (“not negative at all”) to 5 (“very negative,” similar to scales used in Studies 1 and 2), and how difficult they imagined it would be to follow the instruction and viewing the picture, on a scale from 1 (“not difficult at all”) to 5 (“very difficult”). Each trial consisted of the following: the instruction to be used with the imagined picture (5 s), a verbal description of the picture to be imagined and the instruction (10 s), the question, “How negative would you feel after viewing the image and following the instruction?” (8 s), and the question, “How difficult would the task be?” (8 s).

### RESULTS

#### Negativity

**FIGURE 4:**
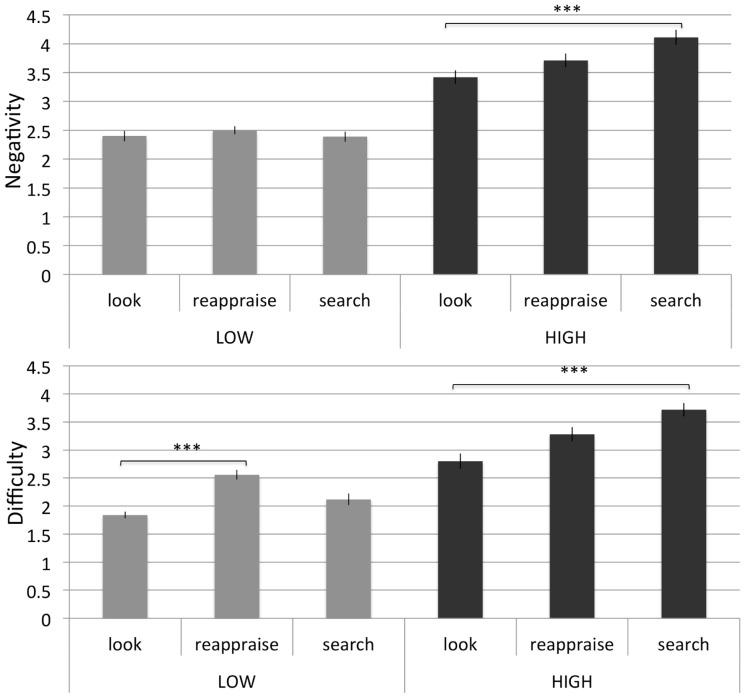
Study 3: mean of negativity and difficulty ratings in low and high imagined negative pictures in the three conditions (“look,” “reappraise,” and “search”), *N* = 41. *p <0.05, **p <0.01, ***p <0.001.

Negativity scores were subjected to a 2 × 3 repeated measurement ANOVA to analyze main effects, as well as the interaction between intensity and instruction type. A main effect of intensity was found [*F*(1,40) = 137.85, *p* <0.001] such that negativity ratings were higher when imagining high intensity pictures (*M* = 3.74, SD = 0.62) than low intensity pictures (*M* = 2.43, SD = 0.66). This result is important because it suggests that the imagination of different intensities was successful in influencing ratings in the expected direction. Mauchly’s test indicated that the assumption of sphericity had been violated for the main effect of instruction [χ^2^(2) = 7.36, *p* = 0.025]; therefore degrees of freedom were corrected using Greenhouse-Geisser estimates of sphericity (ε = 0.83). The main effect of instruction was significant [*F*(1.71,68.27) = 3.91, *p* = 0.031]. These main effects were qualified by a significant interaction [*F*(2,80) = 3.19, *p* = 0.046] in that the instruction conditions affected negativity ratings dependent on the intensity of the picture descriptions. An inspection of the means suggested that under high intensity regulatory “search” (*M* = 4.11, SD = 0.97) resulted in the *highest* expected negativity followed by regulatory “reappraise” (*M* = 3.71, SD = 1.05) and the control “look” condition (*M* = 3.41, SD = 0.94). Follow up comparisons (Bonferroni corrected) revealed that the only significant effect was that under high (but not low) intensity regulatory “search” resulted in significantly *higher* negativity ratings relative to the control “look” condition [*F*(1,80) = 10.88, *p* = 0.004, see **Figure 4**]. These results suggest that participants expect that the Search strategy would be least effective under high intensity, a result that is opposite to Study 2 findings that were obtained while participants actually employed the different conditions.

#### Difficulty

Difficulty scores were subjected to a 2 × 3 repeated measurement ANOVA to analyze the interaction between intensity and instruction type. There was a main effect of intensity [*F*(1,40) = 99.79, *p* <0.001] such that imagining high intensity negative pictures (*M* = 2.18, SD = 0.63) while employing the instructions was more difficult than imagining low intensity pictures (*M* = 3.74, SD = 0.62). There was also a main effect of instruction type [*F*(2, 80) = 11.70, *p* <0.001]. The main effects were qualified by a significant interaction [*F*(2, 80) = 5.76, *p* = 0.005] such that the instruction conditions affected difficulty ratings dependent on the intensity of the pictures. Follow up comparisons (Bonferroni corrected) revealed that in the high intensity condition, regulatory “search” (*M* = 3.72, SD = 0.97) was rated as significantly *more* difficult than the control “look” condition [*M* = 2.80, SD = 1.05, *F*(1,80) = 35.7, *p* <0.001], and regulatory “reappraise” [*M* = 3.28, SD = 1.04, *F*(1,80) = 7.97, *p* = 0.043, see **Figure 4**]. In the low intensity condition, regulatory “reappraise” (*M* = 2.56, SD = 0.97) was rated as significantly more difficult than the control “look” condition [*M* = 1.85, SD = 0.88, *F*(1,80) = 12.05, *p* = 0.002], while regulatory “search” was not significantly different than either the control “look” condition nor regulatory “reappraise.” These results complement the negativity results in showing that participants’ expectations regarding the difficulty of the “search” option are opposite to actual implementation. It also bears noting that expected results regarding reappraisal are also opposite to those reported in Study 2.

## GENERAL DISCUSSION

Cognitive emotion regulation strategies involve exerting executive control processes that can change emotional information processing at an attentional deployment or semantic meaning stages. These two broad regulation categories can be further divided to sub-strategies that vary on the degree of overlap in their underlying cognitive operation, which leads to differential efficacy. Despite a continuum of possibilities, prior studies concentrated on attentional deployment and semantic meaning strategies that had minimal underlying operation overlap because they varied in the type and degree of conflict between regulatory and emotional processes. The major goal of the present study was to test the relative efficacy of a novel attentional deployment sub-strategy (visual search distraction) that has a matched degree of conflict with a classic semantic meaning sub-strategy (reappraisal) under low and high negative intensities.

Based on our conceptual framework (Sheppes and Gross, 2011, 2012) we hypothesized and found that visual search distraction was as effective as cognitive reappraisal in down-regulating the experience of low intensity of negative emotion (Study 1), but more effective and less effortful than cognitive reappraisal when regulating high intensity (Study 2). Underlying mechanisms of visual search distraction revealed that its operation is partially hindered by negative intensity, and that it blocks emotional information from being processed and remembered relative to reappraisal (Study 2). A final study demonstrated that the relative efficacy results obtained with self-reports cannot be fully explained with demand characteristics, because participants’ expectations about how they should feel diverged from how they actually felt while implementing visual search distraction (Study 3).

Our finding that visual search distraction can be more efficacious than reappraisal under high intensity extends our conceptual framework (Sheppes and Gross, 2011, 2012) in important ways. Our framework argues that disengaging early from emotional information processing via attentional deployment strategies (e.g., with distraction) can more successfully block high intensity information relative to engaging with emotional information processing prior to a late modulation (e.g., with reappraisal). However, prior empirical support for our framework comes from studies that contrasted an attentional deployment strategy (distraction) that is different from a semantic meaning strategy (reappraisal) in the type of conflict (attention selection conflict in distraction vs. semantic meaning conflict in reappraisal) and degree of conflict (minimal in distraction and strong in reappraisal) between regulatory and emotional processes. In the present study, we were able to show that differential efficacy in favor of attentional deployment is maintained under high intensity, even when the degree of conflict between the regulatory and emotional processes resembled the high level of conflict in reappraisal. These findings suggest that the basic difference in the type rather than the degree of conflict between attentional deployment and semantic meaning determines strategies’ outcome.

An increase in degree of conflict was operationalized in the present study as the stimulus based competition between the regulatory and emotional processes. A conceptual continuum of regulatory options that vary on this feature can be made. Minimal stimulus based competition is evident when distraction involves a production of unrelated neutral thoughts (e.g., Sheppes and Meiran, 2007, 2008) or during cognitive load maintenance in working memory (e.g., McRae et al., 2010; Van Dillen et al., 2013). Low stimulus based competition is evident when attention and accordingly perception is fixed at a single non-emotional part of an emotional stimulus (e.g., Dunning and Hajcak, 2009; Urry, 2010). Intermediate stimulus based competition is evident when the encoding but not maintenance of cognitive load is occurring simultaneously with picture presentation (e.g., Kanske et al., 2011; Schönfelder et al., 2013). Last, high stimulus based competition is evident in our visual search distraction that involves disengaging from emotional processing by actively scanning multiple subtle non-emotional features that are embedded in potent emotional stimuli. Indeed in the present study we found performance based evidence for stimulus based competition in showing that visual search performance is impaired when emotional intensity is high relative to low. The disengagement feature of visual search distraction was observed in impaired memory of emotional information relative to engagement reappraisal.

Like any other regulatory option visual search distraction can be viewed as a strategy that has a cost-benefit profile. It may be beneficial because it can serve as a crutch in times when our cognitive resources are used up or when facing high intensity situations. However, because the emotional stimulus is not processed, it may lack the long term benefit that requires making sense of emotional events in order to adapt (see Ayduk and Kross, 2008; Thiruchselvam et al., 2011). This particular cost can prove maladaptive and function as a risk or maintaining factor in psychopathology. Specifically, several anxiety disorders entail a tendency to over-generalize a disengagement or avoidance from emotional information processing (see Foa and Kozak, 1986; Campbell-Sills and Barlow, 2007 for reviews). Disengagement usually starts in response to focused high intensity emotional situations, but if overly used it can constrain healthy functioning and lead to maladaptive anxiety response. Disengagement can also contribute to maintenance of existing anxiety disorders, because it does not allow one to question the validity of high intensity fears.

On average individuals who used visual search distractions reported it was relatively simple to implement and that it resulted in feeling less negative. Although, these findings were obtained with self reports, Study 3 helped minimizing demand characteristics effects. Specifically, we found that participants’ expectations about how they should feel diverged from how they actually felt while implementing visual search distraction. This finding suggests that individuals are not fully aware of the potential benefits of employing visual search distraction. While interesting this feature may lead to non-optimal emotion regulation choice that is important for healthy adaptation (see Sheppes and Levin, 2013; Sheppes, 2014, for reviews).

This study showed the effectiveness of an innovative emotion regulation strategy, but several improvements could be made in the future. Although visual search and classic attentional distraction (e.g., Sheppes and Meiran, 2007) appear to behave similarly when compared to reappraisal and thereby confirm and extend the model by Sheppes and Gross (2011), future studies should further investigate the role of the degree of conflict in determining regulatory effectiveness. One option is to parametrically change the degree of conflict in visual search distraction by manipulating the salience of the embedded letters in the visual search. A second option is to compare visual search distraction to classic attentional selection distraction. The advantage of both of these options is that they hold the regulatory attentional selection category constant which would allow testing more directly whether differential degree of conflict affects regulatory effectiveness.

A second limitation is that the preset results were obtained for only one type of emotional inducing stimuli namely emotional pictures. Although, our focus on visual search distraction required using visual stimuli, our data precludes us from knowing whether attentional selection strategies that involve enhanced degree of conflict would be effective with other types of emotional stimuli.

## Conflict of Interest Statement

The authors declare that the research was conducted in the absence of any commercial or financial relationships that could be construed as a potential conflict of interest.
